# Supplementary material for: Mapping the evolution of fertility support policies in China: A content and instrumental analysis
Source: PLoS One. 2025 Oct 9;20(10):e0332137. doi: 10.1371/journal.pone.0332137 (PMC12510515; doi:10.1371/journal.pone.0332137)
Supplement: S1 Appendix — (ZIP) [file pone.0332137.s001.zip › S1 Appendix. 226 original policy documents/210-第十届全国人民代表大会第五次会议关于2006年国民经济和社会发展计划执行情况与2007年国民经济和社会发展计划的决议(FBM-CLI-1-89366).doc]

第十届全国人民代表大会第五次会议关于2006年国民经济和社会发展计划执行情况与2007年国民经济和社会发展计划的决议

**发布部门：** [全国人民代表大会](https://www.pkulaw.com/law/chl?Aggs.IssueDepartment=101)

**发布日期：**2007.03.16

**实施日期：**2007.03.16

**时效性：** [现行有效](https://www.pkulaw.com/law/chl?Aggs.TimelinessDic=01)

**效力级别：** [工作文件](https://www.pkulaw.com/law/chl?Aggs.EffectivenessDic=XA0105)

**法规类别：** [机关工作综合规定](https://www.pkulaw.com/law/chl?Aggs.Category=00301) [人大议事](https://www.pkulaw.com/law/chl?Aggs.Category=00106)

第十届全国人民代表大会第五次会议关于2006年国民经济和社会发展计划执行情况与2007年国民经济和社会发展计划的决议
（2007年3月16日第十届全国人民代表大会第五次会议通过）

　　第十届全国人民代表大会第五次会议审查了国务院提出的《关于2006年国民经济和社会发展计划执行情况与2007年国民经济和社会发展计划草案的报告》及2007年国民经济和社会发展计划草案，同意全国人民代表大会财政经济委员会的审查结果报告。会议决定，批准《关于2006年国民经济和社会发展计划执行情况与2007年国民经济和社会发展计划草案的报告》，批准2007年国民经济和社会发展计划。

关于2006年国民经济和社会发展计划执行情况与2007年国民经济和社会发展计划草案的报告
　　--2007年3月5日在第十届全国人民代表大会第五次会议上
　　国家发展和改革委员会

各位代表：
　　受国务院委托，现将2006年国民经济和社会发展计划执行情况与2007年国民经济和社会发展计划草案提请十届全国人大五次会议审议，并请全国政协各位委员提出意见。
　　一、2006年国民经济和社会发展计划执行情况
　　2006年，全国各族人民在中国共产党的领导下，认真贯彻落实科学发展观和构建社会主义和谐社会重大战略思想，按照十届全国人大四次会议审议批准的国民经济和社会发展计划，加快推进改革开放和现代化建设，发展条件进一步改善，发展环境进一步优化，发展势头进一步趋好，实现了“十一五”良好开局，朝着全面建设小康社会的宏伟目标又迈出了坚实的一步。
　　（一）国民经济运行保持增长速度较快、经济效益较好、物价水平较低的良好态势
　　经济平稳较快发展。全年国内生产总值20.94万亿元，增长10.7%。经济发展的稳定性显著增强，国内生产总值增速年度之间、季度之间波幅都比较小。全年居民消费价格总水平上涨1.5%，在经济增长连续四年达到或略高于10%的同时，物价涨幅稳定在较低水平，这在我国发展史上是少有的。
　　图一：国内生产总值增长率

　　经济效益较好。全国财政收入3.93万亿元，增长24.3%；规模以上工业企业实现利润18784亿元，增长31%。单位国内生产总值能耗下降1.23%，这是2003年以来的首次下降；单位工业增加值用水量下降9%。
　　经济运行中的一些突出矛盾和问题有所缓解。投资增幅有所回落，全年全社会固定资产投资增长24%，增幅比上半年回落5.8个百分点，比上年下降2个百分点。货币信贷增幅趋缓，年末广义货币供应量（M（2下标））同比增长16.9%，增速比5月末回落2.1个百分点，比上年末下降0.6个百分点。煤电运紧张状况明显改善，年末直供电厂存煤2411万吨，处于正常水平；新增电力装机1亿千瓦，年发电量28344亿千瓦时，增长13.4%，国家电网系统拉限电条次和损失电量分别比上年减少97%和82%；粮食、化肥等重点物资运输得到较好保障。
　　（二）社会主义新农村建设扎实推进
　　惠农政策进一步完善。在全国范围内取消了农业税和农业特产税，加上已经取消的屠宰税、牧业税等涉农税费，每年减轻农民负担约1250亿元。实行对种粮农民的直接补贴、良种补贴、农机具购置补贴和农资综合直补，全年补贴超过300亿元；增加对财政困难县和产粮大县的转移支付，奖励补助235亿元。粮食最低收购价范围由稻谷扩大到小麦。较大幅度提高了大中型水利水电工程建设征收耕地补偿标准。
　　支农投入持续增加。中央财政用于“三农”的支出达3397亿元（不包括用石油特别收益金安排的对种粮农民综合直补120亿元），比上年增加422亿元。农村生产生活条件得到改善。农村饮水安全工程建设，中央投入60亿元，比上年增长2倍，地方投入约69亿元，使2897万农民受益；农村沼气建设，中央投入25亿元，比上年增长1.5倍，地方投入15.6亿元，新增沼气用户约450万户；农村公路改造工程，中央安排投资190亿元，地方投入240亿元，共改扩建通乡通村沥青（水泥）路12万公里。实施“万村千乡”市场工程，建成农家店10万多个。
　　图二：农村饮水安全工程建设情况

　　图三：农村沼气建设情况

　　农业结构继续优化。粮食种植面积比上年增加110万公顷，达到10538万公顷。粮食再获丰收，总产量49746万吨，比上年增加1344万吨。优质专用小麦和优质水稻种植面积比重达到55.2%和69.1%，分别比上年提高6个和4个百分点。棉花和糖料产量分别增长17.8%和16.2%；肉类、奶、水产品分别增长4.6%、18%和2.8%。
　　（三）经济结构调整出现积极变化
　　工业结构调整力度加大。规模以上工业中，高技术产业增加值增长18.7%，比工业增幅高2.1个百分点。大型薄板热连轧成套设备、大型电站空冷系统等重大技术装备自主化取得实质性突破。产能过剩行业结构调整取得新进展。依法淘汰了一批落后生产能力，钢铁行业关闭了一批100立方米以下的小高炉和15吨以下的小转炉；电解铝自焙槽已全部淘汰；水泥湿法窑大部分已停产或拆除，新型干法水泥比重超过50%；煤炭行业关闭小矿井5931处，淘汰不符合安全生产条件或破坏资源的落后产能1.1亿吨。
　　图四：2003年以来高技术产业增加值及其新增额情况

　　自主创新能力建设积极推进。出台了一批支持自主创新的配套政策实施细则，十六个重大科技专项相继启动前期论证。全年国家财政科技支出1260.38亿元，比上年增长26.2%。超声医疗、燃料电池等国家工程研究中心组建完成，新增国家认定企业技术中心92家，首批创新型企业试点工作开始启动。下一代互联网示范工程、龙芯2号高性能通用芯片研制等获得重大创新成果，建成首个全超导托卡马克核聚变实验装置。
　　服务业稳步发展。规模继续扩大，增加值达到82703亿元，增长10.3%。结构和质量进一步改善。交通运输供给能力明显增强，新建公路9.37万公里，其中高速公路4325公里；新建铁路投产里程1605公里，增建复线705公里；新扩建沿海港口码头泊位252个，其中万吨级及以上深水泊位144个；首都机场、上海浦东机场改扩建等工程进展顺利，西藏林芝等5个中西部支线机场投入使用。物流配送、金融保险、信息服务等生产性服务业发展加快，消费性服务业中新兴业态、新兴产品不断涌现。
　　区域协调发展取得新成绩。西部大开发“十一五”规划编制完成，全年投到西部地区的中央预算内投资和国债资金比重达41%，年初确定的12项重点工程全部开工建设，特色优势产业发展加快，巩固退耕还林成果，西部五省区基本口粮田建设试点起步。东北地区等老工业基地资源型城市经济转型试点、采煤沉陷区治理和棚户区改造进展顺利，重点企业自主创新和生产制造能力增强。中部崛起战略启动实施，出台了促进中部地区崛起的若干意见，明确了扶持政策。东部地区继续率先发展，天津滨海新区、上海浦东新区综合配套改革试点积极推进。
　　（四）社会事业和民生工作得到加强
　　优先发展教育取得新进展。全国又有83个县通过“两基”验收。农村义务教育经费保障机制改革开局良好，2006年已经惠及5200万名农村中小学学生，平均每个小学生年减负140元，初中生减负180元。中西部农村地区3730万名贫困家庭学生获得免费教科书，780万名农村寄宿学生获得生活费补助。安排中央投资40亿元，支持了2858所寄宿制学校和8.58万所中小学现代远程教育设施的建设。中央投资28.5亿元，支持了321个职业教育实训基地、28所示范性高等职业院校、478所中等职业学校和县职教中心建设。高中阶段教育毛入学率达到59%，高等教育毛入学率达到22%。
　　图五：2003年以来全国实现“两基”目标的县数及其新增数情况

　　公共卫生体系建设和人口计划生育工作继续加强。安排国债资金27亿元，地方配套17亿元，支持了5436所乡镇卫生院和672所县医院、县中医院、县妇幼保健机构的基础设施建设。安排国债资金，在中西部地区建设了一批食品药品监管设施。覆盖全国大中城市的社区卫生服务网络初步形成。参加新型农村合作医疗的农民已有4.1亿人，以县为单位的覆盖率达到50.7%。人口自然增长率5.28‰。
　　图六：以县为单位的新型农村合作医疗覆盖率情况

　　文化、旅游和体育事业加快发展。50户以上通电自然村农民收听收看广播电视的目标全部实现，农村电影放映工程进展顺利。国家图书馆二期、中国美术馆二期和部分省级博物馆等重点文化项目建设稳步推进，文化遗产保护进一步加强。国内旅游、内地公民出境旅游的人次分别增长15%和11.3%，红色旅游点基础设施建设加快。全民健身计划继续推进。北京31个奥运比赛场馆已有30个开工建设。
　　人民群众得到更多实惠。农村居民人均纯收入和城镇居民人均可支配收入分别实际增长7.4%和10.4%。29个省（区、市）提高了最低工资标准。保障农民工工资按时足额支付的各项制度不断完善。城镇新增就业1184万人，年末城镇登记失业率4.1%。社会保障覆盖范围扩大，参加城镇基本养老保险、基本医疗保险、失业保险、工伤保险和生育保险的人数分别增加1162万、1954万、539万、1757万和1038万。25个省（区、市）初步建立了农村最低生活保障制度。各类优抚对象的抚恤补助标准大幅度提高。通过以工代赈在贫困地区新建了一批小型基础设施，安排易地扶贫搬迁试点资金10.6亿元，搬迁西部地区贫困人口24万人。水库移民征地补偿和后期扶持政策逐步完善，受益农民达到2288万人。实施了电力、粮食、化肥、药品、成品油、房地产价格和涉农、涉企、教育、医疗、出租车收费等专项检查。社会消费品零售总额增长13.7%，增幅较上年提高0.8个百分点。
　　图七：2003年以来农村居民人均纯收入及其增长情况

　　图八：2003年以来城镇居民人均可支配收入及其增长情况

　　（五）节能减排和生态保护力度加大
　　节约能源资源和发展循环经济工作明显加强。虽然2006年年初确定的全国节能减排目标没有实现，但各方面对节约能源资源和发展循环经济的认识普遍增强，推动力度加大。发布了[加强节能工作的决定](https://www.pkulaw.com/chl/1f5d4ccc615652a2bdfb.html)，与30个省（区、市）、新疆生产建设兵团和部分中央企业签订了节能减排目标责任书。节能目标责任和评价考核体系初步建立，单位GDP能耗和单位工业增加值水耗公报制度正式实施。全面推进十大重点节能工程，运用国债资金支持了111个重点节能项目，开展千家高耗能企业节能行动，重点耗能企业节能管理得到加强。调整了节能节水产品政府采购清单，强制性能效标识制度实施范围扩大。电力需求侧管理继续加强。对高耗能行业加大实施差别电价政策力度，一些地方还制定了能耗超限额加价政策。广泛开展节能环保宣传，全社会节能环保意识显著增强。组织实施了第一批循环经济试点。
　　环境保护和生态建设积极推进。清洁生产全面展开，发布了国家重点行业清洁生产技术导向目录和评价指标体系。开展淮河、松花江等流域城镇水污染治理和工业废水治理。城市污水处理率和城市生活垃圾无害化处理率达到56%和54%，分别比上年提高4个和2.3个百分点。又有1.04亿千瓦火电机组安装了脱硫设施。全国二氧化硫、化学需氧量的排放量增幅分别下降11.3个和4.4个百分点。全年退耕还林26.7万公顷，退牧还草1000万公顷。
　　图九：2003年以来城市污水处理率和生活垃圾无害化处理率情况

　　图十：2003年以来退耕还林、退牧还草情况

　　（六）改革开放迈出新步伐
　　重点领域和关键环节改革继续深化。北京、内蒙古等8个省份和部分市县开展了农村综合改革试点。19家国有独资公司进行了建立和完善董事会试点，第二批74家中央企业分离办社会职能改革基本完成。垄断行业管理体制改革积极推进，邮政管理初步实现政企分开。中国银行、中国工商银行成功上市，农村金融组织的市场准入门槛进一步降低，银行业资产质量和资本充足率继续提高，上市公司股权分置改革基本完成，直接融资规模扩大，外汇管理制度和人民币汇率形成机制进一步完善。政府收支分类改革顺利推进，预算管理制度改革不断深化。调整和完善了消费税、资源税和房地产税收政策，提高了个人所得税起征点。投资体制改革积极推进，已有30个省（区、市）出台了企业投资项目核准和备案管理办法，80%以上的企业投资项目实行了备案制，中央投资管理程序进一步规范。资源性产品和要素价格形成机制改革取得积极进展，石油价格综合配套改革和电价调整方案顺利实施，30个省（区、市）开征了水资源费，15个城市实行了居民生活用水阶梯式计量水价制度，建立了工业用地出让最低价标准统一公布制度。深化医药卫生体制改革工作开始启动。实施中小企业成长工程，非公有制经济发展环境继续改善。
　　积极推进外贸增长方式转变。进出口总额达1.76万亿美元，比上年增长23.8%。进出口商品结构进一步优化，机电产品、高新技术产品出口分别增长28.8%和29%，自主性强、增值率高的一般贸易出口比重提高，部分高耗能、高污染产品出口过快增长势头得到有效抑制。全年实际吸收外商直接投资695亿美元。企业“走出去”取得新成效，全年对外直接投资（非金融部分）达161亿美元，比上年增长31.6%。
　　经济社会发展在取得成绩的同时，也面临不少困难和挑战。从深层次看，经济结构不合理、增长方式粗放、体制机制不完善等问题还没有根本解决。从当前看，经济运行虽然朝着宏观调控的预期方向发展，但基础还不稳固，经济增长付出的代价过大，不稳定不协调的因素较多，比较突出的有：一是农业基础薄弱的状况没有根本改变，农民持续增收难度加大。农业抗灾能力不强，科技水平不高。粮食生产效益相对较低，对种粮农民补贴的增收效应减弱，农民外出务工收入增加受多种因素影响，制约农民增收的一些体制性障碍尚未消除。二是投资反弹并引发经济波动的风险依然存在。投资增幅仍居高位，在建规模依然偏大，新开工项目较多，导致投资增长过快的体制机制问题还没有根本解决，一些地方、领域投资扩张的动力仍然很强。银行体系流动性过剩仍将持续，贷款扩张能力和放贷意愿较强。外贸顺差继续扩大。三是节能减排形势严峻，资源环境矛盾日趋尖锐。2006年年初确定的节能减排目标未能完成，主要原因是：产业结构调整进展缓慢，服务业比重较低，重工业特别是一些高耗能、高污染行业增长依然偏快，很多本应淘汰的落后能力还没有退出市场；相关法律法规、标准和管理制度还不完善、不配套，节能减排投入不足，支持节能减排的财税、价格、金融等政策措施尚不到位；不少企业开展节能环保技术改造的动力不强，一些节能环保项目建成并发挥作用还需要一定时间；从严执法做得不够，违法成本低，浪费资源、破坏环境等违法违规行为屡禁不止；一些地区对节能环保重要性和紧迫性的认识还有待提高，存在着重经济增长、轻环境保护的现象。2007年节能减排工作任务仍相当艰巨。煤电油运形势虽有所改善，但区域性、时段性、结构性供求紧张状况依然存在。四是社会问题仍较突出，促进社会和谐任务艰巨。就业形势依然严峻，劳动力总量过剩与结构性短缺并存，大学生就业压力增加，下岗失业人员特别是“4050”人员、“零就业家庭”再就业问题依然突出。社会保障覆盖范围较窄。群众对上学难上学贵、看病难看病贵反映强烈，食品安全、药品安全、居民住房、收入分配、社会治安、安全生产等问题仍比较突出，在企业改制、城市拆迁、土地征收征用、环境保护等方面也存在一些损害群众利益的问题，老少边穷地区脱贫任务还很艰巨。对上述问题，要积极采取措施，认真加以解决。

　　二、2007年经济社会发展的总体要求和主要目标
　　2007年的经济社会发展工作，要以邓小平理论和“三个代表”重要思想为指导，认真贯彻党的十六大和十六届三中、四中、五中、六中全会精神，全面落实科学发展观，加快构建社会主义和谐社会，继续加强和改善宏观调控，着力调整经济结构和转变增长方式，着力加强资源节约和环境保护，着力推进改革开放和自主创新，着力促进社会发展和解决民生问题，推动经济社会发展切实转入科学发展的轨道，努力实现国民经济又好又快发展，为党的十七大召开创造良好环境。
　　按照上述要求，兼顾需要和可能，并与“十一五”规划《纲要》相衔接，提出了2007年经济和社会发展的主要目标：
　　--在优化结构、提高效益、降低消耗、减少排放的基础上，保持适当的经济增长速度。第三产业和高技术产业比重提高；财政收入44065亿元；国内生产总值增长8%左右。近几年，国内生产总值增速都在10%或略高一些，今年经济增长的预期目标适当留有余地，是向社会传递政府的调控意图，引导各方面切实把工作重点和注意力放到优化经济结构、转变增长方式、提高质量效益上来，防止片面追求和盲目攀比速度，实现经济又好又快发展。经济增长目标是预期性、指导性的，各地也应从实际出发，合理确定本地区生产总值的增长速度。
　　--城镇新增就业不低于900万人，城镇登记失业率控制在4.6%以内。今年就业形势虽然比较严峻，但随着经济持续较快增长和促进就业政策效应的进一步显现，这一目标通过努力是可以实现的。
　　--城镇居民人均可支配收入和农村居民人均纯收入分别实际增长6%，社会消费品零售总额增长12%。随着国家财力不断增强，企业效益继续提高，收入分配制度改革，以及建设社会主义新农村、促进农民增收、加强社会保障和扶贫等各项措施的落实，城乡居民收入将持续增加。但城镇低收入居民和农民的收入增长仍面临许多困难和制约因素，实现这一目标需下相当大的气力。
　　--居民消费价格总水平涨幅控制在3%以内。主要考虑是：积极稳妥地推进资源性产品价格改革，调整公共产品和服务价格，以及上年底粮油等农副产品价格上涨的滞后影响等，会在一定程度上推动价格总水平上升。
　　--科技、教育、卫生、文化等各项事业进一步发展。研究与试验发展经费支出占国内生产总值的比重提高到1.56%。高中阶段教育毛入学率达到65%，普通高等学校计划招生567万人，研究生42.4万人。公共文化服务体系建设进一步加强。新型农村合作医疗试点范围扩大到全国80%以上的县（市、区）。人口自然增长率控制在7.5‰。
　　--国际收支不平衡状况改善。当前，国际环境总体有利，国内企业及其产品的竞争力进一步提高，出口增速总体上将保持较高水平；同时，随着加入世贸组织过渡期的结束，鼓励进口和服务贸易的政策效应逐步显现，进口规模会适当扩大，贸易顺差增长过快的势头将在一定程度上得到抑制。

　　三、2007年经济社会发展的主要任务和措施
　　实现今年经济和社会发展目标，要统筹安排，突出重点，着力抓好以下八方面工作。
　　（一）继续加强和改善宏观调控，保持经济平稳运行。坚持实施稳健的财政政策和货币政策。（1）适当减少中央财政赤字，继续调整财政支出结构。2007年，中央财政赤字拟控制在2450亿元，比上年预算赤字减少500亿元。合理安排财政超收收入。狠抓增收节支，提高财政资金使用效益。（2）缩减长期建设国债发行规模，合理安排中央投资。建议2007年发行长期建设国债500亿元，比上年减少100亿元；拟安排中央预算内经常性建设资金804亿元，比上年预算增加250亿元；两项合计，中央政府建设投资总规模为1304亿元。按照优化结构、突出重点、加强薄弱环节、集中力量办大事的要求，做到中央政府建设投资用于直接改善农村生产生活条件的投入、用于支持基础教育和公共卫生等社会事业发展的投入、用于西部大开发的投入均高于上年。同时加大对节能环保和自主创新的支持。（3）保持货币供应量合理增长，着力优化信贷结构。2007年，广义货币供应量（M（2下标））预期增长16%。综合运用多种货币信贷工具，继续加强银行体系流动性管理，引导金融机构把握信贷投放节奏，合理控制中长期贷款规模。严格限制对高耗能、高污染企业和产能过剩行业劣质企业的贷款，加大对“三农”、中小企业、自主创新、节能环保、社会事业和欠发达地区的金融支持力度。（4）保持价格总水平基本稳定。加强对价格总水平和粮食、棉花、石油、煤炭、钢材、商品房等重要商品和服务价格走势的监测分析。做好市场价格巡查工作。
　　继续加强固定资产投资调控。（1）严把土地、信贷闸门和市场准入门槛，继续控制产能过剩和消耗高、污染重行业的发展。根据不同行业的情况，适当提高并严格执行建设项目在土地、环保、节能、技术、安全等方面的准入标准。巩固新开工项目清理成果，把好新上项目的市场准入关，特别要控制城市建设规模，严格控制行政机关新建、扩建办公大楼，严禁建设豪华楼堂馆所。建立新开工项目管理的部门联动机制，研究制订新开工项目报告和公开制度。（2）加强关系经济社会全局和长远发展的重大项目建设，支持农业、服务业、社会事业、节能环保、自主创新、欠发达地区等薄弱环节。运用补助、贴息、税收、价格等经济手段，发挥政府投资导向作用，合理引导社会投资。（3）强化投资项目监管，抓紧建立项目前期储备制度，加强项目稽察和监督检查，确保资金安全和工程质量。
　　着力扩大消费需求。（1）加快调整国民收入分配格局，促进城乡居民收入进一步增加，关键是不断提高农民和城镇低收入者的收入水平和消费能力。（2）扩大农村消费需求，加大对农村商品流通网络、农产品批发市场及粮食现代物流等流通基础设施建设的支持力度，完善农资流通网络，继续实施“万村千乡”、“双百”市场工程。（3）落实好房地产市场调控的各项政策，加快建设廉租房和中低价位、中小套型普通商品住房，改进和规范经济适用住房制度，改善低收入家庭住房条件。依法打击房地产开发、交易、中介等环节中的违法违规行为，抑制房地产价格过快上涨。（4）积极拓宽消费领域，大力发展电子商务，积极发展红色旅游、乡村旅游、文化、健身、体育、休闲等消费，培育和扩大消费热点。（5）进一步改善消费环境，完善医疗收费和药品价格政策，规范医疗器械价格，全面推行学校收费公示制度；开展涉农、涉企、教育、医疗、房地产、电信、水电油气等价格和收费的监督检查，加强市场监管，维护消费者合法权益。
　　做好经济运行调节工作。重点建设大型现代化煤矿，整合改造中小型煤矿。加快开发油气资源，稳步构建石油储备体系，加快油气管网设施的规划建设。加强电网建设，优化发展火电，积极开发水电，加快发展核电和太阳能、生物质能、风能、地热等可再生能源。加大煤运通道薄弱环节、下水港口和重大交通基础设施的建设力度。搞好各种交通运输方式综合协调，合理配置交通资源，提高交通运输业综合运营效率。进一步规范有序用电，开展能效电厂试点，推动建立电力需求侧管理长效机制。继续加强煤电油运供需衔接，优先协调解决居民生活需要，保障医院、学校、金融、粮食、化肥等重点领域和单位的需求。
　　（二）坚持以发展农村经济为重点，扎实推进社会主义新农村建设。巩固、完善、加强支农惠农政策，切实加大对“三农”的投入，大力促进农业和农村经济结构调整。（1）稳定发展粮食生产。继续搞好优质粮食产业工程和国家大型商品粮基地建设。完善对种粮农民的直接补贴、农资综合直补政策，扩大良种补贴和农机具购置补贴资金规模。继续对重点地区、重点粮食品种实行最低收购价政策。建立和完善粮食安全预警系统，加强对粮食生产、库存及进出口的监测和调控。（2）积极推进现代农业建设。改进农业基础科研设施和农业技术装备，大力推广农业先进适用技术。加强棉花、油料、糖料生产基地建设，鼓励有条件的地方发展养殖小区，继续实施种养业良种工程、植保工程和现代农业示范项目。搞好农产品质量安全检验检测体系、动物防疫体系建设，健全社会化服务体系。（3）继续改善农村生产生活条件。加快实施沃土工程，加强中小型农田水利设施建设，支持大型灌区节水改造及中部地区排涝泵站建设，加大病险水库的改造力度。增加农村水气路电设施建设的投入，今年再解决3200万农村人口的饮水安全问题，新增农村沼气用户500万户，支持县乡公路改造和通村道路建设，抓好中西部农村电网改造和无电地区电力建设。（4）加快发展农村社会事业。全部免除农村义务教育阶段学生的学杂费。继续对贫困家庭学生免费提供教科书，补助寄宿生生活费。完成农村中小学现代远程教育工程和西部“两基”攻坚任务，继续实施农村寄宿制学校建设工程，启动中西部农村初中校舍改造。加强以乡镇卫生院为重点的农村卫生服务体系建设，扩大新型农村合作医疗覆盖范围。启动乡镇综合文化站建设，推进新一轮广播电视村村通和农村电影放映工程。（5）拓宽农民增收渠道。积极发展农村二、三产业，加快发展高效经济作物和畜牧水产业，提高农产品质量安全水平，努力增加种养业、林业收入，推进农业产业化经营，支持龙头企业发展。发展农村合作经济组织，壮大县域经济。大力开展各类职业技能培训，提高广大农民的就业和创业能力。（6）构建农村投入稳定增长机制。进一步整合支农投资，建立支农投资规划计划衔接和部门沟通机制，完善投入管理办法。
　　（三）着力做好节能减排工作，加强环境保护和节约集约用地。为了实现“十一五”规划《纲要》确定的五年单位国内生产总值能耗降低20%左右、主要污染物排放总量减少10%的目标，要知难而上，采取更有效的措施，做出更大的努力，坚持以节能降耗、污染减排和节约集约用地为突破口和重要抓手，加快经济结构调整和增长方式转变，大力发展服务业和高技术产业，鼓励用先进技术改造传统产业，引导企业兼并重组，加快推动高耗能、高污染行业中不符合准入条件的落后生产能力退出市场，促进集约发展。与此同时，切实抓好以下八方面工作：（1）落实目标责任。严格执行节能降耗和污染减排目标责任制，加快建立科学统一的节能减排统计指标体系、监测体系，健全节能减排工作实绩评价考核机制。（2）突出重点领域。在钢铁、有色、电力、石油石化、化工、建材等重点行业和领域，利用中央和地方投资，全面实施低效燃煤工业锅炉（窑炉）改造、区域热电联产、余热余压利用、节约和替代石油、绿色照明等十大重点节能工程。加强年耗能万吨标准煤以上重点企业节能管理，深入开展千家企业节能行动，加强能源审计，在重点耗能行业选择一批节能潜力大、应用面广的重大节能技术，加大攻关和推广力度。（3）严把源头关口。实行固定资产投资项目节能评估和审查制度，把能耗作为项目审批、核准和开工建设的强制性门槛。实行耗能产品最低能效标准，制定并实施产品能耗限额，建立公共建筑能耗定额制度，扩大强制性能效标识实施范围。改进发电调度方式，加快火电行业“上大压小”的工作进度，今年要关停1000万千瓦小火电机组。所有新建火电厂都要同步安装脱硫设施，发布实施现有燃煤电厂二氧化硫治理规划，组织开展烟气脱硫特许经营试点。力争淘汰落后炼铁产能3000万吨、落后炼钢产能3500万吨。（4）强化政策支持。完善有利于节能降耗的财税政策，中央财政设立节能专项资金，支持高效节能产品推广、重大节能项目建设和重大节能技术示范。完善高耗能、高污染行业差别电价和水价政策，健全排污收费制度，年底前所有城镇都要开征污水处理费，并逐步提高收费标准，所有设市城市都要在规定期限内开征垃圾处理费。提高安装脱硫设施的燃煤电厂上网电价，对非正常停运脱硫设施的电厂要扣减脱硫电价，并从严处罚。出台鼓励利用余热余压、煤矸石和垃圾发电及上网电价优惠政策。（5）发展循环经济。抓紧编制国家循环经济发展规划，完善循环经济评价指标体系和统计制度，推进第一批循环经济试点。积极开展资源综合利用，做好再生资源回收利用、废旧家电回收处理和汽车零部件再制造试点工作。（6）加强环境保护。全面推行清洁生产，对严重超标排放企业实施强制性清洁生产审核，限期完成清洁生产改造。继续做好“三河三湖”、渤海、松花江、三峡库区及上游、南水北调水源及沿线等重点流域和区域污染综合防治，加强城市饮水安全保障，提高城市污水和垃圾处理能力，依法取缔饮用水源保护区内的直接排污口。大力发展环保产业。（7）搞好监督检查。开展节能减排专项检查，加强对节能减排目标责任制、能效标识和高耗能企业节能措施等落实情况的监督检查。对重点耗能企业和重点污染源实施在线监测，加大对已建成的城镇污水、垃圾处理和脱硫设施运行情况的监督。（8）节约集约用地。实行最严格的土地管理制度，认真执行土地利用总体规划和年度计划，切实加强新增建设用地总量控制，保护耕地特别是基本农田，禁止擅自将农用地转为建设用地。严格执行建设用地定额指标和工业用地出让最低价标准，规范土地出让收支管理，国有土地使用权出让收支全额纳入地方政府性基金预算。
　　（四）大力增强自主创新能力，推动产业结构优化升级。（1）认真落实国家中长期科技发展规划纲要，再出台一批支持自主创新的配套政策实施细则。组织实施国家重大科技专项，建设一批重大科技基础设施，组建一批国家工程实验室和国家工程研究中心，选择支持一批国家认定的企业技术中心，推动应用技术研究院所与生产企业重组。继续建设公共技术支撑平台，加强技术咨询、技术转让等中介服务，加大知识产权保护力度。（2）加快发展服务业。搞好服务业重点领域规划和项目建设。放宽和规范行业准入，运用财税、价格等政策，支持服务业关键领域、薄弱环节和新兴行业发展。积极吸收国际先进经验、技术和管理方式，提升服务业的质量和水平。（3）调整优化工业结构。落实促进产业结构调整暂行规定和[产业结构调整指导目录](https://www.pkulaw.com/chl/2c6d5a70e784ea19bdfb.html)，完善行业准入管理制度，加快产能过剩行业结构调整，鼓励和支持钢铁、水泥、电解铝、煤炭、纺织、造纸等行业的企业兼并重组，依法淘汰落后生产能力。密切关注其他行业生产和市场供求变化，及时采取措施，防止出现新的产能过剩。广泛应用先进信息技术改造传统产业，特别是要加强对钢铁、建材、煤炭、电力、石油化工、建筑等行业的技术改造。（4）增强高技术产业的核心竞争力。继续实施生物医药、集成电路、新一代移动通信、下一代互联网、信息安全等高技术工程和产业化专项，加快出台软件和集成电路、数字电视、生物等产业政策。新建电子信息、生物和航空航天等高技术产业基地，培育一批新兴产业群。（5）大力振兴装备制造业。尽快出台鼓励购买和使用国产首台（套）重大技术装备的办法，制定高档数控机床、基础制造装备重大专项规划和总体实施方案。落实岭澳二期、红沿河核电项目关键设备自主化，实施1000千伏交流输变电、800千伏直流输变电项目试验示范工程，巩固发展城市轨道交通装备自主化的成果，推进高技术船舶以及船用设备自主化，加快大型造船基础设施建设。（6）有序发展替代能源。抓紧研究制订替代能源中长期发展规划以及煤基替代能源、生物质能等专项规划，先行试点，稳步推进，防止不顾条件一哄而起。
　　（五）落实区域发展总体战略，促进区域经济协调发展。（1）继续推进西部大开发。组织实施西部大开发“十一五”规划，加快基础设施、生态环境建设和科技教育发展。新开工建设一批重点工程，加强西部铁路、干线公路、支线机场和内河航运基础设施建设。抓好优势特色资源开发和产业基地建设。加快制定相关后续政策，巩固和发展退耕还林、退牧还草成果。继续实施天然林保护、防沙治沙、石漠化治理等重点生态工程。加强西部人才开发和法制工作。加大东中西协调互动力度。（2）积极推动东北地区等老工业基地振兴。支持企业联合重组和重大装备研发制造，推动装备制造、原材料和农副产品加工等产业的优化升级，加强松嫩、三江平原商品粮基地建设，继续做好资源枯竭型城市经济转型试点，推进采煤沉陷区治理和棚户区改造。（3）落实促进中部崛起的政策措施。加强中部粮食基地、能源原材料基地、先进制造业和高技术产业基地以及综合交通运输体系建设，加快发展现代流通业。提高对外开放水平，有序承接东部地区和国外产业转移。（4）鼓励东部地区不断推动体制机制创新，加快产业结构优化升级，在推进改革开放、促进科学发展、转变增长方式方面走在全国前列。落实天津滨海新区开发开放的相关政策措施，积极推进上海浦东新区、深圳经济特区等综合配套改革试点。（5）加快革命老区、民族地区、边疆地区和贫困地区的经济社会发展，加大对欠发达地区基础设施建设和公共服务的支持力度，积极扶持人口较少民族的发展。（6）分层次开展主体功能区规划的编制工作。编制完成国家主体功能区规划，扎实推进省级主体功能区规划的编制。（7）积极稳妥地推进城镇化。重点是提高城市综合承载能力，优先发展公共交通，着力解决大城市交通拥挤、环境污染等突出问题，加快“城中村”改造。（8）做好海洋规划，扶持海洋经济发展。
　　（六）继续深化各项改革，提高对外开放质量和水平。加强总体指导和统筹协调，建立健全体制改革协调推进机制，努力在一些重点领域和关键环节的改革上取得突破。（1）积极推进行政管理体制改革。科学界定政府机构职责，健全行政运行机制，改进政府管理与服务方式，推进依法行政，强化行政问责制。（2）全面深化农村改革。巩固和发展农村税费改革成果。着力推进以乡镇机构、农村义务教育和县乡财政管理体制改革为主要内容的农村综合改革。稳定和完善农村土地承包关系，保障农民权益。探索发展适合农村特点的新型金融组织，积极培育多种形式的小额信贷组织，提高农村金融服务水平。（3）继续推进企业改革。加快国有大型企业股份制改革，健全公司法人治理结构、投资风险控制机制和内部监督管理机制，进一步做好国有企业政策性关闭破产、分离办社会职能和主辅分离工作，建立国有资本经营预算制度。深化电信、电力、民航、邮政管理体制改革，加快推进铁路投融资体制改革，研究提出铁路体制改革总体方案，继续推进供水、供气、供热等市政公用事业改革。全面落实鼓励、支持和引导非公有制经济发展的政策措施。继续实施中小企业成长工程，健全中小企业社会化服务体系。（4）加快金融改革与创新。稳步有序推动中国农业银行改革。积极推进政策性银行以及其他商业银行、金融资产管理公司和非银行金融机构改革。大力发展资本市场和保险市场，构建多层次金融市场体系，加强债券市场基础性制度建设，扩大企业债券发行规模，做好产业投资基金试点工作。稳步推进利率市场化改革，完善人民币汇率形成机制。健全金融监管体系，防范和化解金融风险。（5）稳步推进财税体制改革。完善财政转移支付制度，深化预算管理制度改革，制定全面实施增值税转型方案及配套措施，健全矿产资源有偿使用制度，加快建立生态环境补偿机制。（6）深化投资体制改革。改进企业投资项目核准程序，颁布项目申请报告示范文本，修订《政府核准的企业投资项目目录》，统一规范企业投资项目备案制，继续推行代建制，抓紧建立政府投资决策责任追究制度。（7）完善资源性产品价格形成机制。逐步理顺成品油和天然气价格，适时调整各类水价标准，加快输配电价改革步伐，完善风能、生物质能等可再生能源发电价格和费用分摊政策，推进供热价格改革。（8）加快转变外贸增长方式。引导拥有自主知识产权和核心技术的产品出口，提高一般贸易和高附加值产品出口比重，控制高耗能、高污染和资源性产品出口，扩大能源、重要矿产品以及先进技术装备、关键零部件进口，加强重要资源的战略储备。提高加工贸易企业准入门槛，推动加工贸易转型升级。（9）以引进先进技术、管理经验和高素质人才为重点，提高利用外资质量。积极稳妥地推进服务业对外开放，积极引导外商投资向中西部、东北地区等老工业基地和符合产业政策的领域转移，加大对中西部地区借用国外优惠贷款的支持力度。完善促进境外投资的政策措施和协调机制，鼓励和规范企业的对外投资合作。积极参与多边贸易规则制定和自由贸易区建设。
　　（七）大力发展教育、卫生、文化事业，进一步增强经济社会发展的协调性。（1）坚持优先发展教育事业。推动公共教育资源向农村、中西部地区、贫困地区、边疆地区、民族地区倾斜。落实好农村义务教育经费保障新机制，解决好城市困难家庭和农民工子女接受义务教育的问题。加强职业教育基础能力建设，重点支持面向农村学生的中等职业教育。以提高质量为核心，加快高等教育教学改革，继续实施高等教育“211”工程，搞好高等院校重点学科建设。建立健全国家奖学金、助学金制度，落实助学贷款政策，在教育部直属师范大学实行师范生免费教育。（2）完善公共卫生和基本医疗服务体系。逐步建立覆盖城乡居民的基本卫生保健制度。健全城市社区卫生服务体系，落实经费保障措施，方便群众防病治病，减轻医疗费用负担。继续做好重大传染病防治工作。大力扶持中医药和民族医药发展。抓紧研究提出深化医药卫生体制改革的总体思路和政策措施。（3）大力发展文化、广播影视、新闻出版、体育等事业。积极推进公共文化服务体系特别是农村文化基础设施的建设，加强抢救性文物和文化自然遗产地保护，加快国家博物馆、国家图书馆二期、中国美术馆二期等重大文化工程建设，推进文化体制改革。全力搞好奥运场馆建设。（4）稳定现行生育政策和低生育水平，提高出生人口素质。全面推行农村计划生育家庭奖励扶助制度，改善农村基层计划生育服务条件，扩大“少生快富”试点范围。大力发展老龄事业。
　　（八）认真解决涉及人民群众切身利益的突出问题，促进和谐社会建设。（1）继续实施积极的就业政策。大力发展就业吸纳力强的劳动密集型产业、中小企业、服务业。加强就业培训，提高劳动者的就业技能。重点做好高校毕业生就业和下岗失业人员再就业工作，积极帮助“零就业家庭”和就业困难人员就业。全面推行劳动合同制度。开展就业促进和统筹城乡就业试点。（2）切实做好收入分配工作。适当提高初次分配中劳动报酬的比例。健全工资协商机制，合理调整并严格执行最低工资制度，进一步规范国有企事业单位特别是垄断行业的收入分配。加强税收征管，有效调节过高收入。实施工资支付保证金制度，强化农民工工资支付监管，切实维护农民工合法权益。（3）加快社会保障体系建设。尽快使城镇基本养老、基本医疗和工伤保险覆盖到各种所有制企业，稳步扩大做实基本养老保险个人账户试点工作，开展以大病统筹为主的城镇居民基本医疗保险试点，大力推进农民工工伤保险和大病医疗保障工作。研究社会保险关系跨地区转移接续办法。健全社会保险基金、住房公积金管理制度，确保资金安全。进一步完善城市低保制度。在全国建立农村最低生活保障制度，合理确定低保对象范围和标准，中央财政对困难地区给予适当补助。加快完善城乡社会救助体系，加强对残疾人、农村“五保户”和孤残儿童等的救助和保护。（4）强化食品药品安全和安全生产工作。继续开展食品药品安全专项整治，大力加强食品药品质量和餐饮卫生监管。完善安全生产体制机制，强化企业安全生产主体责任，积极推进煤矿瓦斯治理和整顿关闭两项攻坚任务。组织实施危险化学品、烟花爆竹、交通运输、消防、建筑、民爆器材等重点行业安全专项整治，加强学校和娱乐场所的安全防范。（5）继续做好扶贫开发工作。管好用好财政扶贫资金和以工代赈资金，加强国家扶贫开发工作重点县农村小型基础设施建设，继续进行易地扶贫搬迁试点。落实水库移民后期扶持政策。认真做好灾区恢复重建工作。（6）维护和促进社会稳定。高度重视并认真解决土地征收征用、房屋拆迁、企业改制、涉法诉讼等方面的问题。加强应急保障，健全应急预案体系。加强社会治安防控体系建设，广泛开展平安创建活动。
　　各位代表，2007年改革发展稳定的任务艰巨而繁重。我们要在以胡锦涛同志为总书记的党中央领导下，高举邓小平理论和“三个代表”重要思想伟大旗帜，全面落实科学发展观和构建社会主义和谐社会重要战略思想，齐心协力，扎实工作，奋发进取，为实现经济和社会发展目标，促进国民经济又好又快发展而努力奋斗！
　　附件：

　　名词解释

　　2006年西部大开发新开工重点工程。2006年西部大开发新开工建设12项重点工程，总投资 1654亿元，包括：太原至中卫铁路、公路建设、支线机场建设、重点煤矿工程、水电站建设、云南青山嘴水库、退耕还林配套基本口粮田、四川80万吨乙烯、新疆罗布泊120万吨钾肥、内蒙古40万吨氧化铝、高技术产业项目、教育卫生等社会事业项目。
　　农村义务教育经费保障机制改革。2005年底，国务院决定深化农村义务教育经费保障机制改革，逐步将农村义务教育全面纳入公共财政保障范围，建立中央和地方分项目、按比例分担的农村义务教育经费保障机制。主要内容包括：全部免除农村义务教育阶段学生学杂费，对贫困家庭学生免费提供教科书并补助寄宿生生活费；提高农村义务教育阶段中小学公用经费保障水平；建立农村义务教育阶段中小学校舍维修改造长效机制；巩固和完善农村中小学教师工资保障机制。上述内容均明确了不同地区的中央与地方财政分担比例，中央重点支持中西部地区，适当兼顾东部部分困难地区。2006年改革在西部地区和中部试点地区实施，2007年将覆盖全国农村地区。
　　新型农村合作医疗。新型农村合作医疗制度是相对二十世纪八十年代以前传统农村合作医疗模式而言，由政府组织、引导、支持，农民自愿参加，个人、集体和政府多方筹资，以大病统筹为主的农民医疗互助共济制度。为实现到2010年在全国建立基本覆盖农村居民的新型农村合作医疗制度的目标，2003年在全国选择了304个县 (市)先行进行试点。主要任务是，研究和探索适应经济发展水平、农民经济承受能力、医疗服务供需状况的新型农村合作医疗政策措施、运行机制和监管方式，为全面建立新型农村合作医疗制度提供经验。
　　水库移民后期扶持政策。国家自2006年7月 1日起，实行统一的水库移民后期扶持政策，扶持范围为建国以来所有大中型水库农村移民，扶持标准为每人每年600元，扶持期限为20年。所需资金主要通过在全国范围内提高省级电网销售电价筹措(扣除农业生产用电)，分省安排使用。此外，国家还将通过其他渠道筹措资金，加大扶持力度，解决库区和移民安置区基础设施和长远发展问题。
　　十大重点节能工程。为落实《节能中长期专项规划》，国家发展和改革委员会组织编制了《“十一五”十大重点节能工程实施方案》，包括：节约和替代石油工程、燃煤工业锅炉(窑炉)改造工程、区域热电联产工程、余热余压利用工程、电机系统节能工程、能量系统优化工程、建筑节能工程、绿色照明工程、政府机构节能工程和节能监测(服务)体系建设工程。“十一五”期间，通过实施十大重点节能工程，预计可节能2．4亿吨标准煤，减排二氧化碳(以碳计)1．56亿吨。
　　强制性能效标识制度。是依据国家有关法律法规实施的一项节能管理制度，即由国家制定并公布统一的产品目录、产品能效标准、能效标识实施规则、能效标识样式和规格。2005年3月1日，国家发展和改革委员会和国家质检总局联合制定的《[能源效率标识管理办法](https://www.pkulaw.com/chl/4cd041b37a7a9267bdfb.html)》正式实施，要求指定的用能产品必须加施符合相关标准要求的信息标签，明示产品的能效等级、能源消耗量等能效性能指标。
　　工业用地出让价最低标准统一公布制度。为加强对工业用地的调控和管理，促进土地节约集约利用，[国土资源部发出《关于发布实施<全国工业用地出让最低价标准>的通知](https://www.pkulaw.com/chl/97215d1876697476bdfb.html)》，从2007年 1月1日起实施。《通知》规定，工业用地出让最低价标准与土地等别挂钩，其中最高等别(一等)相对应的最低价标准为840元／平方米，最低等别(十五等)相对应的最低价标准为60元／平方米。
　　银行体系流动性。我国银行体系流动性由金融机构在中央银行的超额存款准备金和金融机构持有的库存现金构成，是金融机构创造货币的基础。影响银行体系流动性的因素通常包括法定存款准备金率、中央银行公开市场操作、再贴现、再贷款、外汇占款、财政在中央银行存款以及流通中现金等。
　　“万村千乡”市场工程。是指2005年开始实施的农村现代流通网络建设工程。国家通过安排财政资金，以补助或贴息的方式，引导城市连锁店和超市等流通企业向农村延伸发展“农家店”力争用三年时间(2005年-2007年)，在全国建设25万家标准化“农家店”，覆盖75％以上的县，形成以城区店为龙头、乡镇店为骨干、村级店为基础的农村现代流通网络，以改善农村消费环境，满足农民生产生活需求。
　　“双百”市场工程。是指2006年开始实施的农产品市场建设专项工程。国家通过安排财政资金，以补助或贴息的方式，重点改造100家大型农产品批发市场、培育100家大型农产品流通企业，达到构建与国际市场接轨的农产品现代流通体系、保障农产晶消费安全、拓宽农民持续增收渠道的目的。
　　农资综合直补政策。是指在现行粮食直补制度基础上，对种粮农民因柴油、化肥、农药等农业生产资料增支实行的综合性直接补贴政策。补贴规模根据预计全年农业生产资料价格变动对农民种粮收益的影响综合测算确定，并对柴油调价硬性增支因素予以充分考虑。补贴资金全部由中央财政负担，一次性拨付给地方并重点向粮食主产区和产粮大县倾斜，年内不再随后期农业生产资料实际价格变动而调整。2006年共安排农资综合直补120亿元。
　　农村寄宿制学校建设工程。为落实《国家西部地区“两基”攻坚计划》，国务院决定在2004-2007年实施农村寄宿制学校建设工程。工程实施范围以截至2002年底尚未实现“两基”的西部 372个县、新疆生产建设兵团的38个团场为重点，并兼顾中部省份部分困难地区，包括约7700所学校。建设内容以农村寄宿制学校的教学用房、学生生活服务用房和教学辅助用房的建设和改造为主。截至2006年底，已下达中央专项资金90亿元，建设学校7651所，预计可满足新增学生195万人、新增寄宿生207万人的就学和寄宿需求。
　　能源审计。是指用能单位自己或委托从事能源审计的机构，根据国家有关节能法规和标准，对能源使用的物理过程和财务过程进行检测、核查、分析和评价的活动。
　　可再生能源发电价格和费用分摊政策。指为鼓励发展风能、生物质能、太阳能、海洋能和地热能等可再生能源发电项目而实行的电价政策。可再生能源发电项目上网电价实行政府定价或者通过招标确定，高于当地脱硫燃煤机组标杆上网电价的部分，在全国省级及以上电网销售电量中分摊解决。
　　供热价格改革。指根据合理补偿成本、合理确定收益、促进节约用热、坚持公平负担的定价原则制定和调整供热价格，推动城市供热由按面积计费逐步过渡到按用热量计费，以促进供热行业发展和节约用热。同时，将对居民采暖供热的补贴由暗补改为明补。
　　城镇居民基本医疗保险试点。城镇居民基本医疗保险是国家面向未纳入城镇职工基本医疗保险制度覆盖范围的学生、儿童和其他非从业城镇居民建立的基本医疗保险制度，采取个人和家庭缴费为主、财政对困难群体给予适当补助的筹资办法，重点保障住院和门诊大病等医疗支出。城镇居民基本医疗保险与城镇职工基本医疗保险、新型农村合作医疗制度共同构成我国覆盖城乡全体居民的基本医疗保险制度体系。国家将从2007年开始，选择若干城市开展城镇居民基本医疗保险试点。

第十届全国人民代表大会财政经济委员会关于
　　2006年国民经济和社会发展计划执行情况与
　　2007年国民经济和社会发展计划草案的审查结果报告
　　(2007年3月12日第十届全国人民代表大会第五次会议主席团第二次会议通过)

十届全国人大五次会议主席团：
　　第十届全国人民代表大会第五次会议审查了国务院提出的《关于2006年国民经济和社会发展计划执行情况与2007年国民经济和社会发展计划草案的报告》以及2007年国民经济和社会发展计划草案。全国人民代表大会财政经济委员会在对计划报告和计划草案进行初步审查的基础上，根据各代表团和有关专门委员会的审查意见，又作了进一步审查。国务院根据审查意见对报告作了修改。现将审查结果报告如下。

　　一、财政经济委员会认为，2006年，国务院和地方各级人民政府认真落实党中央确定的方针政策和十届全国人大四次会议的决议，国民经济和社会发展计划执行情况是好的，实现了“十一五”的良好开局。宏观调控和结构调整取得成效，经济平稳快速增长，经济效益稳步提高，物价基本稳定，粮食连续三年增产，社会主义新农村建设顺利推进，重点领域的改革继续深化，对外开放迈出重要步伐，自主创新能力不断提高，就业和社会保障工作取得新进展，城乡人民生活继续改善，各项社会事业加快发展，我国的综合国力进一步增强，我们朝着全面建设小康社会的宏伟目标又迈出坚实的一步。同时，在经济和社会发展中也存在不少问题和困难。一二三产业结构不合理，城乡之间、地区之间发展不平衡，粮食稳定增产和农民持续增收难度加大，节能降耗和污染减排的年度目标没有实现，投资增长过快、信贷投放过多、国际收支不平衡的矛盾还比较突出，食品药品安全、就业、医疗、教育、住房、收入分配、安全生产等方面还存在不少问题。这些都需要在今后工作中继续努力解决。

　　二、财政经济委员会认为，2007年国民经济和社会发展计划报告和计划草案体现了“十一五”规划纲要和中央经济工作会议的精神和要求，突出了着力调整经济结构和转变增长方式、着力加强资源节约和环境保护、着力推进改革开放和自主创新、着力促进社会发展和解决民生问题的工作重点，指导思想明确，主要目标和总体安排可行。财政经济委员会建议批准国务院提出的《关于2006年国民经济和社会发展计划执行情况与2007年国民经济和社会发展计划草案的报告》，批准2007年国民经济和社会发展计划草案。

　　三、财政经济委员会认为，完成2007年国民经济和社会发展计划，要按照落实科学发展观和构建社会主义和谐社会的要求，着重抓好以下工作。
　　（一）加强和改善宏观调控，促进经济又好又快发展。继续实施稳健的财政政策和货币政策，保持宏观经济政策的连续性和稳定性，并根据经济运行的新情况及时完善政策措施，保持经济平稳运行。切实控制投资过快增长，继续严把土地、信贷闸门，提高建设项目在环保、节能、安全等方面的准入标准，严格控制新上项目和城市建设规模，遏制重复建设，提高投资效益。着力扩大消费需求，提高消费对经济增长的拉动作用。综合运用多种经济政策手段，有效缓解国际收支顺差过多问题。引导银行控制信贷投放，重点控制中长期贷款增长，加大对“三农”、中小企业和欠发达地区的金融支持。要在政策落实上狠下功夫，确保宏观调控各项措施落到实处。
　　（二）巩固农业基础地位，扎实推进社会主义新农村建设。继续坚持把解决好“三农”问题作为重中之重，统筹城乡经济社会发展。建立稳定长效机制，继续加大对“三农”投入力度。切实加强农村基础设施建设，增加水气路电设施建设投入，特别要重视中小型农田水利建设。加强对支农资金的整合与监管，提高使用效益。大力发展现代农业，推进农业和农村经济结构调整，切实提高农业质量、效益和竞争力。加大对粮食主产区的扶持力度，稳定发展粮食生产，确保粮食安全。完善各项惠农支农政策，多渠道增加农民收入。全面提高农民素质，培养造就新型农民，发挥农民在社会主义新农村建设中的主体作用。
　　（三）加大节能减排工作力度，扭转环境质量恶化的趋势。节能减排目标是“十一五”规划纲要提出的重要约束性指标，必须加大工作力度，坚定不移地实现。要进一步提高对节能减排工作重要性的认识，以节能降耗、保护环境和集约用地为切入点，促进结构调整、增长方式转变和质量、效益提高。推进节能环保法律法规的制定修订工作，健全产品能耗和环保标准，加强和完善相关统计工作。严格落实目标责任，强化监督和问责机制，严肃查处违法行为。完善矿产资源有偿使用制度，加快形成有效的资源开发和生态补偿机制。充分发挥市场的调节作用，完善土地、能源、水等资源产品价格形成机制，使资源价格能够反映市场供求、稀缺程度和生态环境成本。鼓励、支持开发利用新能源和可再生能源。积极推进清洁生产，大力发展循环经济。采取切实有力措施，加大重点流域区域污染防治力度，做好城市废气、污水、固体废物污染防治工作。
　　（四）着力调整经济结构，切实提高自主创新能力。调整优化产业结构，采取有效政策措施，加快发展服务业特别是现代服务业，着力提高服务业比重和水平。积极发展高新技术产业，振兴装备制造业，依法坚决淘汰落后生产能力，提升工业层次和水平。落实区域发展总体战略和各项政策措施，发挥各地优势，促进区域经济协调健康发展。加强综合交通运输体系建设，优化交通运输结构。增强自主创新能力，鼓励企业在技术创新中发挥主体作用，健全支持自主创新的财税、金融政策和政府采购制度，完善促进创业风险投资的政策，切实加大知识产权保护力度，加快创新型国家建设。
　　（五）更加注重解决民生问题，积极促进和谐社会建设。下大力气解决人民群众在上学、就业、看病、住房等方面存在的问题，提高中低收入者收入，使广大人民群众共享改革发展成果。始终把教育摆在优先发展的战略地位，普及和巩固义务教育，加快发展职业教育，着力提高高等教育质量。积极促进教育公平，推动公共教育资源向农村、中西部和老少边穷地区倾斜。继续实行积极的就业政策，加强就业服务和培训，加大对困难群体就业的扶持力度。创造良好政策环境，积极支持自主创业和自谋职业。调整国民收入初次分配和再分配格局，规范垄断行业收入分配，加大税收调节力度，合理调整和严格执行最低工资制度。健全完善社会保障体系，进一步扩大社会保障覆盖面。维护农民工合法权益，着力解决农民工养老、医疗、工伤保险和子女接受义务教育等方面的问题。建立和完善农村最低生活保障制度和城市低保制度。尽快建立覆盖城乡居民的基本卫生保健制度，完善公共卫生和医疗服务体系，继续推进新型农村合作医疗制度和城市社区卫生服务体系建设。建立健全廉租房制度，改善低收入家庭住房条件。认真做好食品药品安全工作，抓紧制定相关法律。加强安全生产特别是煤矿安全生产管理，完善突发事件应急处理机制，推进社会治安综合治理，维护社会稳定。
　　（六）深化各项改革，提高对外开放水平。深化国有企业改革，加快建立国有资本经营预算制度，推进垄断行业改革。落实鼓励、支持和引导非公有制经济发展的各项政策和配套措施。继续深化国有商业银行改革，推动政策性银行改革，完善农村金融体系。大力发展资本市场，提高直接融资比重。切实加强和改进金融监管，有效防范化解金融风险，维护国家金融稳定和安全。进一步转变外贸增长方式，优化进出口商品结构，继续控制高耗能、高污染产品出口，扩大进口，妥善应对国际贸易摩擦，努力改善贸易不平衡状况。提高利用外资质量，合理引导外资投向。鼓励和规范企业对外投资合作。积极贯彻实施[行政许可法](https://www.pkulaw.com/chl/6cc71b5743a5ee2abdfb.html)，转变政府职能。努力降低行政成本，厉行节约，反对浪费。
　　以上报告，请予审议。

全国人民代表大会财政经济委员会
　　2007年3月12日

© 北大法宝：（[www.pkulaw.com](https://www.pkulaw.com/)) 专业提供法律信息、法学知识和法律软件领域各类解决方案。北大法宝为您提供丰富的参考资料，正式引用法规条文时请与标准文本核对。
欢迎查看所有[产品和服务](https://www.pkulaw.com/helps/2.html)。法宝快讯：[如何快速找到您需要的检索结果？](https://www.pkulaw.com/helps/70.html)[法宝](https://www.pkulaw.com/helps/69.html)V6有何新特色？

扫描二维码阅读原文

**原文链接：**<https://www.pkulaw.com/chl/2c7837a134cdccfdbdfb.html>
